# Comparative Genomics of Iron-Transporting Systems in *Bacillus cereus* Strains and Impact of Iron Sources on Growth and Biofilm Formation

**DOI:** 10.3389/fmicb.2016.00842

**Published:** 2016-06-08

**Authors:** Hasmik Hayrapetyan, Roland Siezen, Tjakko Abee, Masja Nierop Groot

**Affiliations:** ^1^Laboratory of Food Microbiology, Wageningen UniversityWageningen, Netherlands; ^2^Top Institute of Food and NutritionWageningen, Netherlands; ^3^Microbial Bioinformatics, NIZOEde, Netherlands; ^4^Center for Molecular and Biomolecular Informatics, Radboud University Medical CentreNijmegen, Netherlands; ^5^Wageningen UR Food and Biobased ResearchWageningen, Netherlands

**Keywords:** *Bacillus cereus*, iron transport, genotypes, growth, biofilm formation, complex iron sources

## Abstract

Iron is an important element for bacterial viability, however it is not readily available in most environments. We studied the ability of 20 undomesticated food isolates of *Bacillus cereus* and two reference strains for capacity to use different (complex) iron sources for growth and biofilm formation. Studies were performed in media containing the iron scavenger 2,2-Bipyridine. Transcriptome analysis using *B. cereus* ATCC 10987 indeed showed upregulation of predicted iron transporters in the presence of 2,2-Bipyridine, confirming that iron was depleted upon its addition. Next, the impact of iron sources on growth performance of the 22 strains was assessed and correlations between growth stimulation and presence of putative iron transporter systems in the genome sequences were analyzed. All 22 strains effectively used Fe citrate and FeCl_3_ for growth, and possessed genes for biosynthesis of the siderophore bacillibactin, whereas seven strains lacked genes for synthesis of petrobactin. Hemoglobin could be used by all strains with the exception of one strain that lacked functional petrobactin and IlsA systems. Hemin could be used by the majority of the tested strains (19 of 22). Notably, transferrin, ferritin, and lactoferrin were not commonly used by *B. cereus* for growth, as these iron sources could be used by 6, 3, and 2 strains, respectively. Furthermore, biofilm formation was found to be affected by the type of iron source used, including stimulation of biofilms at liquid-air interphase (FeCl_3_ and Fe citrate) and formation of submerged type biofilms (hemin and lactoferrin). Our results show strain variability in the genome-encoded repertoire of iron-transporting systems and differences in efficacy to use complex iron sources for growth and biofilm formation. These features may affect *B. cereus* survival and persistence in specific niches.

## Introduction

Iron is one of the essential elements required for growth and metabolism of the majority of microorganisms. Despite its important role in microbial cells, the availability of free iron in the environment is limited due to oxidation of ferrous iron to ferric ions which precipitate near neutral pH (Ratledge and Dover, 2000). Free ferrous iron can be toxic to mammals due to formation of oxygen radicals, consequently the majority of host iron is bound to transport molecules such as hemoglobin (red blood cells), transferrin (serum), and lactoferrin (milk and mucosal secretions), or to ferritin-like proteins for intracellular iron storage (Ratledge and Dover, 2000). The storage of iron in complexed form also reduces its availability for invading pathogenic microorganisms. However, many pathogens developed mechanisms to overcome iron scarcity by the expression of scavenging systems specific to complex and non-complex iron sources. Two main scavenging mechanisms for iron have been described. Bacteria may secrete specific molecules with high affinity to iron named siderophores (Ratledge and Dover, 2000; Zawadzka et al., 2009) that facilitate iron transport into the microbial cell. These siderophores sequester iron from different sources such as transferrin (Abergel et al., 2008). The second mechanism involves specific ABC-type transporters encompassing high-affinity surface receptors specific for either complex iron compounds or free iron (Brown and Holden, 2002; Daou et al., 2009). *B. cereus* genomes encode several putative ABC transporters for complexed iron including ferric citrate (Harvie and Ellar, 2005; Fukushima et al., 2012) and ferrichrome, and several others of unknown substrate specificity (Hotta et al., 2010). Furthermore, a possible interplay between different molecules has been suggested. For example the heme-binding surface protein IlsA in *B. cereus* also serves as ferritin receptor and assists in ferritin-iron sequestration by bacillibactin siderophore (Segond et al., 2014). IlsA has also been shown to transfer bound hemin to another surface iron transporting molecule of the IlsA system IsdC (Abi-Khalil et al., 2015).

For *B. cereus, two* different siderophores, bacillibactin (BB), and petrobactin (PB) (Wilson et al., 2006) have been identified. PB is the main siderophore for *B. anthracis* (Koppisch et al., 2005) and important for its virulence since it is not recognized by the innate immune system (Abergel et al., 2006). In *B. cereus*, BB seems to be of higher importance in virulence compared to PB based on experiments in an insect model (Segond et al., 2014).

*B. cereus* has been reported to use various iron sources for growth that are typically present in red blood cells such as hemoglobin (Hb), hemin, and other hemoproteins (Sato et al., 1998, 1999a,b). For *B. cereus* ATCC 14579, the use of ferritin as an iron source has been described (Daou et al., 2009). Concerning the use of transferrin by different *B. cereus* strains, contradictory reports have been published that conceivably links to strain variability (Sato et al., 1998; Park et al., 2005; Daou et al., 2009) and pointing to the importance to take strain diversity into account in studies on iron metabolism. Lactoferrin, an iron source typically present in milk, cannot be used by *B. cereus* and inhibits its growth when present in high concentrations (Sato et al., 1999b; Daou et al., 2009). Ferric citrate, an iron source formed from citric acid which is commonly present in milk and citrus fruits, can also be used by *B. cereus* (Fukushima et al., 2012). These iron sources can be encountered in different environments including soil, food and processing environments, and mammals or insects. The ability to use these sources largely determines the fitness of bacteria and capacity to adapt to specific niches.

Besides its important role as essential element for bacterial growth and virulence (Cendrowski et al., 2004; Harvie et al., 2005; Porcheron and Dozois, 2015), iron has also been reported to affect biofilm formation (Porcheron and Dozois, 2015). It was recently shown that air-liquid biofilm formation by a selection of *B. cereus* food isolates was stimulated by addition of FeCl_3_ (Hayrapetyan et al., 2015a). Biofilm formation may serve as survival mechanism in different environments and can be an important factor contributing to host colonization. To our knowledge, the impact of different (complex) iron sources on biofilm formation capacity and type of biofilms formed including submerged or surface-attached liquid-air biofilms, has not been reported for this species.

In this study we investigated the use of different iron sources by 22 *B. cereus* strains in relation to their genome content. Expression of the iron transporters in iron deplete and replete conditions was studied in the reference strain ATCC 10987. Since the ability of *B. cereus* to form biofilms contributes to its persistence in environment and free iron availability is important for biofilm formation of *B. cereus* (Hayrapetyan et al., 2015a), we also studied the effect of iron sources encountered in different environments on biofilm formation.

## Results

### Iron transporting systems presence and expression

**Figure 1:**
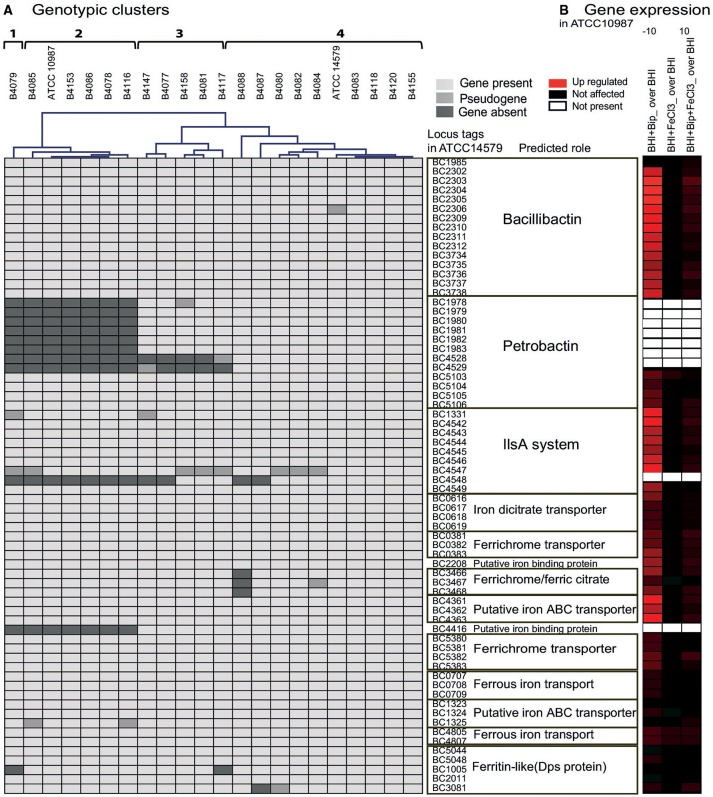
**(A)** Hierarchical clustering of 22 *B. cereus* strains based on gene repertoire encoding iron transporters. Clustering was performed using Genesis software (Sturn et al., 2002). **(B)** Expression of genes encoding iron transporters in *B. cereus* ATCC 10987 in BHI, BHI+Bip, BHI+FeCl_3_, and BHI+Bip+FeCl_3_ at exponential growth phase (*t* = 5 h). Up-regulated genes are presented in red, down-regulated genes in green and unaffected genes in black. The scale −10 to 10 is based on log_2_ values of expression ratios compared to BHI.

Genomes of 20 food isolates and 2 reference *B. cereus* strains ATCC 14579 and ATCC 10987 were analyzed for genes with predicted function in iron transport (Figure 1A and Table 1). Genes encoding for synthesis of siderophore BB structural components (*dhbACBEF*) and transporters were present in all strains, while PB biosynthesis genes (*asbABCDEF*) were absent in seven of the 22 strains analyzed. For five strains, PB biosynthesis genes were present but a functional *fpuA/fhuB* gene cluster necessary for PB uptake was lacking. However, another permease (*fatCD*) with a redundant function with *fhuB* (Dixon et al., 2012), was identified in all the strains in a cluster together with ATP- and substrate-binding proteins (BC5103–5106). Interestingly BC4416, a *fhuD*-like putative iron compound binding protein with unknown specificity (Hotta et al., 2010) was absent in the strains that also lacked PB siderophore biosynthesis genes, which could indicate a role for this protein in PB transport.

**Table 1:** **Genes and their predicted function in iron transport in *B. cereus***.

**Locus tag in *B. cereus* ATCC 14579**	**Locus tag in *B. cereus* ATCC 10987**	**Predicted role**	**Name**	**Predicted function**
BC1985	BCE2066	Bacillibactin	ymfD	Hypothetical protein
BC2302	BCE2398		dhbA/entA	3,3-dihydro-3,3-dihydroxybenzoate dehydrogenase
BC2303	BCE2399		dhbC	Isochorismate synthase
BC2304	BCE2400		dhbE	3,3-dihydroxybenzoate-AMP ligase
BC2305	BCE2401		dhbB	Isochorismatase
BC2306	BCE2402		dhbF	Non-ribosomal surfactin synthetase SrfAA
BC2309	BCE2403		mbtH	Hypothetical protein
BC2310	BCE2404			Drug resistance transporter, EmrB/QacA family
BC2311	BCE2405		sfp	Putative 3′-phosphopantetheinyl transferase
BC2312	BCE2406			Hypothetical protein
BC3734	BCE3767		yuiI	Trilactone hydrolase
BC3735	BCE3768		feuD/yusV	Siderophores ABC-transporter, ATP-binding protein FeuC
BC3736	BCE3769		feuC	Siderophores ABC-transporter, permease FeuC
BC3737	BCE3770		feuB	Siderophores ABC-transporter, permease FeuB
BC3738	BCE3771		feuA	Siderophores ABC-transporter, siderophore-binding protein FeuA
BC1978		Petrobactin	asbA	Petrobactin biosynthesis protein AsbA
BC1979	–		asbB	Petrobactin biosynthesis protein AsbB
BC1980	–		asbC	Acyl-CoA synthetase
BC1981	–		asbD	Acyl carrier protein
BC1982	–		asbE	Petrobactin biosynthesis protein AsbE
BC1983	–		asbF	Hypothetical protein
BC4528	–		fpuA	Iron compound ABC transporter substrate-binding protein
BC4529	–		fhuB	Iron-hydroxamate transporter permease subunit
BC5103	BCE5223		fhuC	Iron-siderophore ABC transporter ATP-binding protein
BC5104	BCE5224		fatC	Iron-siderophore ABC transporter permease
BC5105	BCE5225		fatD	Iron-siderophore ABC transporter permease
BC5106	BCE5226		fatB	Iron-siderophore ABC transporter binding lipoprotein
BC1331	BCE144	IlsA	ilsA	Iron-regulated Leu-rich surface protein A
BC4542	BCE4666			Heme-degrading monooxygenase IsdG
BC4543	BCE4667			Sortase B
BC4544	BCE4668			Iron compound ABC transporter, ATP-binding protein
BC4545	BCE4669			Iron compound ABC transporter, permease protein
BC4546	BCE4670			Iron compound ABC transporter, iron compound-binding protein
BC4547	BCE4671			Iron transport-associated protein
BC4548	–			Iron transport-associated protein, NEAT domain
BC4549	BCE4672		isdC	Iron transport associated protein
BC0616	BCE0683	Iron dicitrate transporter	fhuD	Iron (III) dicitrate ABC transporter, iron compound-binding protein
BC0617	BCE0684		fecD	Iron (III) dicitrate ABC transporter, permease protein
BC0618	BCE0685		fecC	Iron (III) dicitrate ABC transporter, permease protein
BC0619	BCE0686		fecE	Iron (III) dicitrate ABC transporter, ATP binding protein
BC0381	BCE0449	Ferrichrome transporter	fhuG	Ferrichrome ABC transporter, permease protein
BC0382	BCE0450		fhuB	Ferrichrome ABC transporter, permease protein
BC0383	BCE0451		feuA	Ferrichrome ABC transporter, ferrichrome-binding lipoprotein
BC2208	BCE2283	Putative iron binding protein	yfiY	Putative iron compound-binding protein
BC3466	BCE3485	Ferrichrome/ferric citrate	feuA-like	Iron compound ABC transporter substrate-bindingprotein FeuA
BC3467	BCE3486		fhuG-like	Ferrichrome transport system permease fhuG
BC3468	BCE3487		fhuB-like	Ferrichrome transport system permease fhuB
BC4361	BCE4448	Putative iron ABC transporter	fepC-lik	Iron compound ABC transporter, ATP-bindingprotein
BC4362	BCE4449		fhuG-like	Iron compound ABC transporter, permease protein
BC4363	BCE4450		fhuD-like	Lipoprotein binding vitamin B13
BC4416	–	Putative iron binding protein	fhuD-like	Putative iron compound-binding protein
BC5380	BCE5509	Ferrichrome transporter	fepB-like	Iron compound ABC transporter, iron compound-binding protein
BC5381	BCE5510		fepC-like	Ferrichrome ABC transporter ATP-binding protein
BC5382	BCE5511		fhuG-like	Ferrichrome ABC transporter permease
BC5383	BCE5512		fhuB-like	Ferrichrome ABC transporter permease
BC0707	BCE0782	Ferrous iron transport	feoB-C	Ferrous iron transport protein FeoB, C-terminal domain
BC0708	BCE0782		feoB-N	Ferrous iron transport protein FeoB, N-terminal region
BC0709	BCE0783		feoA	Ferrous iron transport protein FeoA
BC1323	BCE1436	Putative iron ABC transporter		Putative iron compound ABC transporter, ironcompound-binding protein
BC1324	BCE1437			ABC transporter ATP-binding protein
BC1325	BCE1438			Iron compound ABC transporter permease
BC4805	BCE4965	Ferrous iron transport	feoB	Ferrous iron transport protein B
BC4807	BCE4966		feoA	Ferrous iron transport protein A
BC5044	BCE5191	Ferritin-like (Dps protein)		Ferritin-like diiron-binding protein, Dps family
BC5048	BCE5196			Ferritin-like diiron-binding protein, Dps family
BC1005	BCE1087			Ferritin-like diiron-binding protein, Dps family
BC2011	BCE2092			Ferritin-like diiron-binding protein, Dps family
BC3081	BCE3134			Ferritin-like diiron-binding protein, Dps family

The IlsA-system acts as a hemophore, and is encoded by the *ilsA* gene (BC1331) and an *isd*-like operon consisting of the ABC-transporter (BC4544-4546), sortase (BC4543), heme degrading monooxygenase (BC4542), and heme transport associated proteins BC4547, BC4548, and BC4549 (IsdC) in *B. cereus* ATCC 14579 (Daou et al., 2009). Genes encoding the IlsA system are present in all strains. In B4079 the IlsA protein appears to be truncated and non-functional due to a point mutation in the encoding gene that creates a premature stop codon. In B4147 the IlsA also appears to be ineffective due to a large internal deletion identified in the encoding gene (both verified with PCR and sequencing). The transport associated protein (BC4547) was identified as a pseudogene in eight strains. Interestingly, the other transport associated secreted component of this system BC4548, which may function as a hemophore that captures heme from Hb and has 98% identity to *isdX1* of *B. anthracis* (Daou et al., 2009), was absent in 11 strains.

Several other known iron ABC-transporters, such as an iron (III) dicitrate-binding complex (*fhuD, fecCDE*), a ferrichrome-binding complex (*feuA/fhuGB*), a *fepC/fhuGD* complex and a *fepBC/fhuGB* complex, were present in all strains. The *feuA/fhuGB* complex, known to bind ferric citrate in *B. cereus* (Fukushima et al., 2012), was only absent in strain B4088. The putative iron-binding protein *yfiY* (BC2208) was identified in all strains. Three additional systems, two of which encode ferrous iron transport FeoB-FeoA proteins (Kim et al., 2012), were identified in all *B. cereus* strains. Besides iron uptake genes, proteins involved in iron storage in bacteria, as for example the ferritin-like di-iron-binding proteins of the Dps family (DNA protection during starvation; Tu et al., 2012) were considered. Five genes with putative function in iron storage were identified and were present in most strains with a few exceptions (Figure 1A). The global regulator of iron uptake Fur (Harvie et al., 2005) was also present in all strains.

Transcriptome analysis of ATCC 10987 in iron replete (BHI+FeCl_3_; BHI+Bip+FeCl_3_) and deplete (BHI+Bip) conditions showed significant upregulation of most of the above mentioned genes encoding iron transporters under iron starvation evoked by addition of the scavenger (Figure 1B).

Iron transport genes were upregulated from 8 up to 900 fold (Supplementary Table 1), which was most prominent for the BB biosynthesis genes. Ferritin-like proteins for storage of intracellular iron were not significantly affected. The second ferrous iron transport cluster FeoA/B (BCE4965-4966) was significantly up regulated during iron starvation, indicating that the so called “living fossil” (Hantke, 2003) might still be functional in atmospheric conditions. Upon supplementation with FeCl_3_, none of these genes were significantly affected, with exception of BCE3769. This was the case also in the presence of Bip together with FeCl_3_ (with BCE2399 as an exception), showing that addition of iron reversed the iron starvation effect of Bip and support a role in iron transport and metabolism for these genes. These results indicate that iron scavenger Bip can be used to assess the efficacy of alternative (complex) iron sources to support growth of the selected 22 strains.

### Iron sources and growth

**Figure 2:**
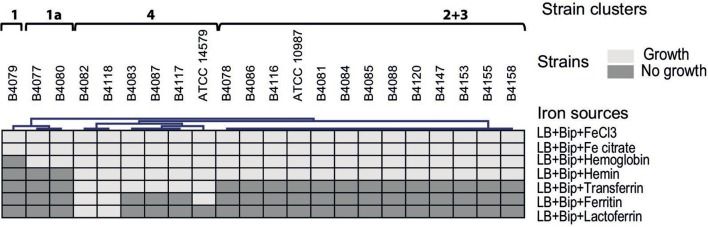
**Phenotypic hierarchical clustering of 22 *B. cereus* strains based on ability to grow on different iron sources**. Strains were clustered using the Genesis software (Sturn et al., 2002).

**Figure 3:**
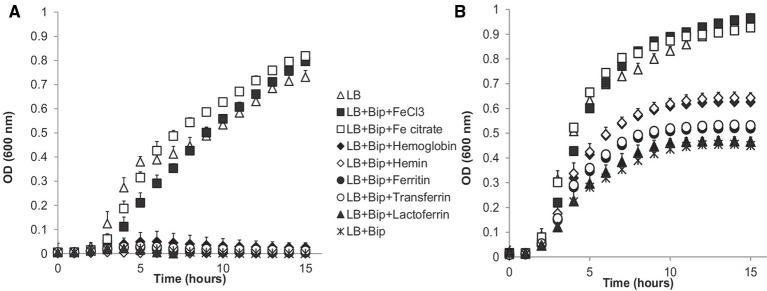
**Growth of strains B4079 (A) and ATCC 14579 (B) in LB and LB supplemented with iron scavenger (LB+Bip) with and without addition of different iron sources**. Presented values are averages of 3 independent experiments with standard deviation. Data points for LB for both strains are very close to FeCl_3_ and Fe citrate. For B4079 **(A)** data points of LB+Bip are very close to LB+Bip+Lactoferrin, LB+Bip+Transferrin, LB+Bip+Ferritin and LB+Bip+Hemin. For ATCC 14579 **(B)** LB+Bip data points are very close to LB+Bip+Lactoferrin; LB+Bip+Ferritin is close to LB+Bip+Transferrin; LB+Bip+Hb is close to LB+Bip+Hemin.

**Figure 4:**
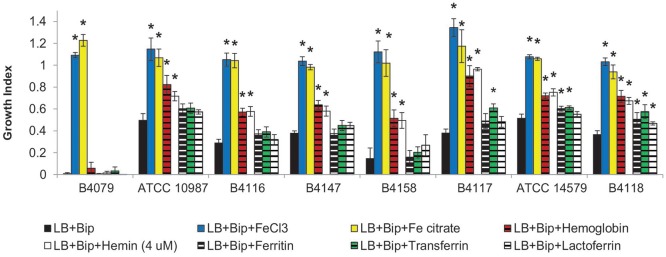
**Growth indexes for selected strains in LB, LB supplemented with iron scavenger (LB+Bip) with and without supplementation with different iron sources**. Growth indexes represent the ratio of OD(600 nm) reached after 10 h of growth with the corresponding iron source relative to the OD reached in LB. Asterix (^*^) indicates significant difference (*p* < 0.01) from iron depleted condition (LB+Bip) for each strain, indicating that the strain could grow with the supplemented iron source.

The ability of *B. cereus* strains to use different iron sources for growth was tested in LB+Bip medium (Figures 2–4). The capacity to cope with iron starvation varied highly among the different strains (Figures 3, 4). Notably, growth of all strains was restored in the presence of either Fe citrate or FeCl_3_ by 80–135% according to growth index (GI) values. All strains, except B4079, could grow with Hb as sole iron source and restored growth to levels ranging from 43% for strain B4078, up to 90% for strain B4117, compared to control conditions (LB medium).

**Figure 5:**
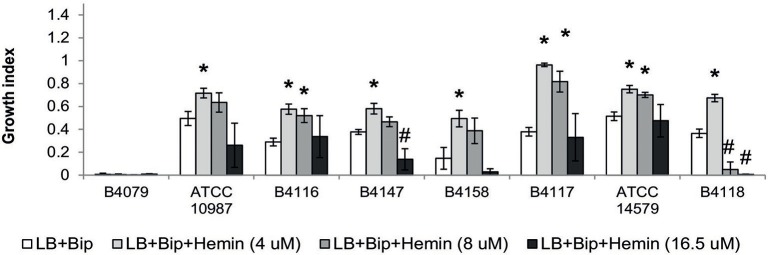
**Growth of selected *B. cereus* strains on different concentrations of hemin**. Growth is expressed as the growth indexes. Asterix (^*^) indicates significant difference (*p* < 0.01) from growth index in LB+Bip for each strain showing that the strain could grow on the specified concentration of hemin, while the hash (#) shows that the growth was significantly inhibited. For all of the presented strains, 4 uM hemin was the optimal concentration for growth, with the exception of strain B4079 which did not grow on this iron source with any concentration.

Hemin could be used by all except three strains (B4077, B4079, B4080). Notably, bacteria that use heme as an iron source also have to cope with its toxicity. This is achieved by a tight control of heme transport, biosynthesis, and degradation. All strains harbored genes to synthesize protoheme and heme, as well as genes encoding the heme efflux ABC transporter HrtA-HrtB, and the associated two-component system HssS-HssR (Stauff and Skaar, 2009; not shown). Only in strain B4158 the latter gene cluster appeared impaired due to an internal deletion, and this strain was among those most sensitive to hemin, along with B4118 and B4147 that were inhibited at higher hemin concentrations (Figure 5).

Transferrin and ferritin could be used by six and three strains, respectively (Figure 2), and both compounds restored growth to a maximum of 60% of the control. Lactoferrin was a poor iron source for most strains and could only be used by strains B4082 and B4118 (Figure 2) albeit that growth was restored to a maximum of 47% of the controls (not shown).

### Linking genotypes with growth phenotypes

The growth performance data on different iron sources and genome contents were clustered (Figures 2, 1A). Four main clusters could be distinguished but phenotypes did not match fully with predicted capacity based on gene content. B4079 showed poorest growth in iron-depleted condition and with complex iron sources. In line with this observation, B4079 lacks most functional transporters. B4079 clusters separately from the other strains (cluster 1, Figures 1A,B) and based on gene content it is most similar to the subgroup of strains lacking PB encoding genes (cluster 2, Figure 1A). The strains of cluster 2 (Figure 1A), along with the strains missing *fpuA/fhuB* genes for PB import (cluster 3, Figure 1A), belong to one large phenotypic cluster (cluster 2+3, Figure 2) of strains which can use FeCl_3_, Fe citrate, Hb, and hemin, but not transferrin, ferritin or lactoferrin. The exceptions are B4077 (no growth on hemin) and B4117 (can use transferrin) which fall out of the phenotypic cluster 2+3. The other five strains that could use more than three of the above mentioned complex iron sources group together based on phenotypes (cluster 4, Figure 2) and they harbor all or most iron transporter genes considered (genotypic cluster 4, Figure 1A). Notably, the other five strains with all the genes present did not match the expected use of complex iron sources. On the other hand, the *feuA/fhuGB* complex is lacking in strain B4088 which nevertheless can grow on Fe citrate. Overall, the phenotypes for 15 out of 22 strains (70%) corresponded to that predicted based on genome content.

### Iron sources and biofilm formation

**Figure 6:**
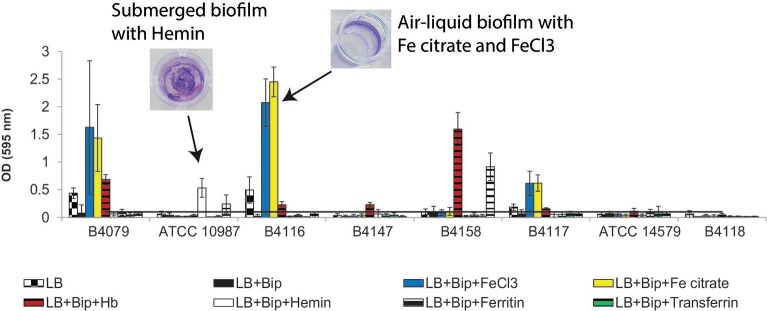
**Biofilm formation for selected *B. cereus* strains**. The biofilm was formed in polystyrene 96-well-plate in LB and LB supplemented with Bip, with or without addition of different iron sources. The biofilm was measured with the CV assay after 24 h incubation at 30°C.

The ability of the different strains to form biofilms with different types of iron sources was tested on polystyrene microtiter plates. 10 out of 22 tested strains formed a biofilm in LB medium without supplementation (control; Table 2). Removal of free iron with Bip eliminated the biofilm forming capacity of nine of these strains, leaving only strain B4155 positive for biofilm formation. For two strains (B4080 and B4120), biofilm formation was promoted under iron deplete condition (Table 2), even though the growth was reduced. Supplementation with Fe citrate and FeCl_3_ not only restored but even increased biofilm forming capacity of the above mentioned 10 strains, and additionally triggered biofilm formation by B4087 (Table 2). Hb allowed biofilm formation by 16 strains, among them 6 strains that did not form biofilm in the control condition, albeit the amount of formed biofilm was lower than that formed in presence of FeCl_3_ or Fe citrate for most of the strains. In the presence of hemin, six strains were able to form biofilm, similar to lactoferrin. These biofilms were completely submerged on the bottom of the well, in contrast to the air-liquid interface biofilm formed in LB, LB+Bip+FeCl_3_, and LB+Bip+Fe citrate (Figure 6).

**Table 2:** **Biofilm formation in the presence of different iron sources**.

**Strains**	**LB**	**LB+Bip**	**LB+Bip+ FeCl_3_ (250 uM)**	**LB+Bip+Fe citrate (250 uM)**	**LB+Bip+ Hemoglobin (2.5 uM)**	**LB+Bip+ Hemin (4 uM)**	**LB+Bip+ Ferritin (0.9 uM)**	**LB+Bip+ Transferrin (1.5 uM)**	**LB+Bip+ Lactoferrin (0.7 uM)**
B4078	+	–	++^*^	++^*^	+^*^	–^*^	–	–	–
B4079	+	–	++^*^	++^*^	+	–	–	–	–
B4082	–	–	–^*^	–^*^	–^*^	–^*^	–^*^	–^*^	–^*^
B4083	++	–	++^*^	++^*^	++^*^	+^*^	–	+^*^	–
B4086	+	–	++^*^	++^*^	+^*^	–^*^	–	–	–
B4087	–	–	+^*^	+^*^	+^*^	–^*^	–	–^*^	–
B4116	+	–	++^*^	++^*^	+^*^	–^*^	–	–	–
B4117	+	–	+^*^	+^*^	+^*^	–^*^	–	–^*^	–
B4118	–	–	–^*^	–^*^	–^*^	–^*^	–^*^	–^*^	–^*^
ATCC 14579	–	–	–^*^	–^*^	+^*^	–^*^	–^*^	+^*^	–
ATCC 10987	–	–	–^*^	–^*^	–^*^	+^*^	–	–	+
B4077	–	–	–^*^	–^*^	+^*^	–	–	–	+
B4080	–	+	–^*^	–^*^	+^*^	+	+	+	+
B4081	–	–	–^*^	–^*^	+^*^	–^*^	–	–	–
B4084	–	–	–^*^	–^*^	–^*^	–^*^	–	–	–
B4085	+	–	++^*^	++^*^	+^*^	–^*^	–	–	–
B4088	–	–	–^*^	–^*^	–^*^	+^*^	–	–	–
B4120	–	+	–^*^	–^*^	–^*^	+^*^	+	+	+
B4147	–	–	–^*^	–^*^	+^*^	–^*^	–	–	–
B4153	+	–	++^*^	++^*^	+^*^	–^*^	–	–	–
B4155	+	+	+^*^	+^*^	+^*^	+^*^	+	+	+
B4158	+	–	–^*^	+^*^	++^*^	–^*^	–	–	+
Total number of biofilm forming strains	10	3	10	11	16	6	3	5	6

*The biofilm was formed in polystyrene 96-well-plates in LB medium, and LB supplemented with Bip with or without addition of indicated iron sources. The biofilm was measured with CV assay after 24 h incubation at 30°C*.

*+, OD (Crystal violet assay) > 0.1*.

*++, OD (Crystal violet assay) > 1*.

–*, OD (Crystal violet assay) < 0.1*.

* *growth was significantly restored compared to LB+Bip*.

*All the positives are highlighted in gray*.

## Discussion

In this study we present data showing the impact of different iron sources on growth and biofilm formation capacity and type of biofilms formed for 20 *Bacillus cereus* food isolates and two reference strains.

Bacillibactin (BB) and petrobactin (PB) are iron-transporting siderophores produced by *Bacillus cereus* group members. The relevance of PB in *B. anthracis* growth and virulence was shown, however for *B. cereus* BB was suggested to be of more importance (Segond et al., 2014). Notably, BB is present in all the strains in this study, while PB is absent in seven strains.

Limitation of free iron impaired the growth of all tested *B. cereus* strains in LB+Bip but was most prominent for B4079, lacking both PB siderophore and functional IlsA. This also prevented efficient use of Hb and hemin by this strain, in contrast to strains missing only one of the mentioned systems. Interestingly, strains able to use ferritin or transferrin as iron source encompass the whole repertoire of iron transporters, with only minor exceptions. This is in agreement with the previously suggested cooperation between different systems such as IlsA and petrobactin siderophore in iron uptake from ferritin (Segond et al., 2014).

The ability of *B. cereus* strains to grow on complex iron sources does not always correspond to the presence of relevant genes. For example, B4120 and B4155 contain the full repertoire of iron transporters, however these strains could not use transferrin, ferritin or lactoferrin as iron sources. This may be explained either by differences in regulation of expression of these genes in the selected conditions, presence of transcriptional activators such as specific iron starvation ECF factors (Visca et al., 2002), or factors that affect translation or activity of the synthesized proteins.

Contradictory data have been reported previously concerning the use of transferrin by *B. cereus*. According to one report, *B. cereus* could use human transferrin as an iron source, albeit with lower efficiency compared to *Staphylococcus aureus, Escherichia coli*, and *Pseudomonas aeruginosa* (Park et al., 2005). Two other studies report inability of *B. cereus* to grow on transferrin (Sato et al., 1998; Daou et al., 2009), or growth inhibition of *B. cereus* and *B. anthracis* by human transferrin (Sato et al., 1998) due to iron deprivation (Rooijakkers et al., 2010). Our data show that the ability to use human transferrin is strain and concentration dependent, concentrations exceeding 2 uM displayed a bacteriostatic effect on several strains (not shown), while 1.5 uM transferrin was the optimal concentration that could be used by 6 out of 22 strains. Besides, the source of transferrin seems of importance since the *S. aureus* transferrin receptor was shown to bind preferentially human and rodent transferrin but not that of bovine and porcine origin (Modun and Williams, 1999). Aerobic or anaerobic growth conditions could also play a role since oxygen availability has for example been shown to affect the relative abundance of petrobactin and bacillibactin in *B. anthracis* (Lee et al., 2011). Furthermore, all the strains used in this study, with the exception of ATCC 14579 (isolated from air in a cow shed) were food isolates. Systemic infections caused by *B. cereus* (Bottone, 2010; Uchino et al., 2012) are caused by more clinically relevant strains, that likely differ in their ability to use and tolerate high levels of transferrin compared to food isolates. To test this, further studies including clinical isolates should be performed.

Lactoferrin is abundant in milk, but also in blood and secreted fluids such as tears and displays antimicrobial properties (Oram and Reiter, 1968; Sato et al., 1999b; Orsi, 2004). Lactoferrin can be used as an iron source by *Pseudomonas* ssp. (Xiao and Kisaalita, 1997) and several other microorganisms (Morgenthau et al., 2013), but not by *B. cereus* as reported previously (Sato et al., 1999b; Daou et al., 2009). The latter study used 1.5 uM of lactoferrin, which in our study also did not restore the growth of any of the 22 strains and inhibited the growth for strain B4086 (not shown). However, a concentration of 0.7 uM lactoferrin slightly restored the growth of two strains (B4082 and B4118), which could also use all other tested iron sources, indicating that these strains were in general better equipped for use of complex iron sources, in line with the full repertoire of iron transporting systems present in these strains. The low number of strains able to use lactoferrin is unexpected given the fact that *B. cereus* is a common contaminant in dairy products.

The capacity to use different complex iron sources could not be linked to the isolation source of the strains. However, clustering of the strains used in this study according to Guinebretière et al. (2008), revealed that all strains lacking petrobactin encoding genes belong to the phylogenetic group III (Warda et al., in press). A common habitat for strains of group III are dehydrated/starchy foods (Guinebretière et al., 2008). Interestingly, all group III strains in the current study were isolated from a starch or dairy containing food product as reported previously (Hayrapetyan et al., 2015a).

### Iron sources and biofilm formation

Previously, we reported that addition of free iron (FeCl_3_) promoted formation of air-liquid interface biofilms by *B. cereus* strains. In this study we show that apart from FeCl_3_ also Fe citrate promoted biofilm formation. Hb triggered biofilm formation for a subset of strains for which the growth was also restored and resulting in partial submerged and air-liquid biofilms. Even strain B4079, which did not show significant growth recovery with Hb, was able to form biofilm upon its addition. It showed very limited growth in the presence of Hb (to *OD* = 0.05, compared to LB+Bip *OD* = 0.01, Figure 3), which may have caused stress conceivably linked to biofilm formation as a response. Hb was previously identified as a component in nasal secretions that promoted colonization by *S. aureus* via repression of the *agr* quorum sensing system resulting in reduced production of proteases with concomitant reduction in biofilm dispersal (Pynnonen et al., 2011). Interestingly this effect was found to be exerted by the Hb protein independently of its iron content. The mechanism of Hb-induced biofilm formation in *B. cereus* remains to be elucidated.

Ferritin and transferrin only slightly supported biofilm formation, mostly for strains already able to form biofilm in iron limited conditions (B4080, B4120, and B4155, Table 2). A role for the surface protein IsdC in cell-cell attachment and biofilm formation under iron deplete conditions was shown for *Staphylococcus lugdunensis* (Missineo et al., 2014). Interestingly, this protein is a homolog of BC4549, encoding a component of the IlsA iron transporting system. Since iron starvation most likely triggers the upregulation of such proteins this may be linked to biofilm-promoting effect of iron depletion for strains B4080 and B4120 (Table 2).

The iron-chelating properties combined with a direct bactericidal effect of lactoferrin has led to its proposed role as potential anti-biofilm compound (Ammons and Copié, 2013). In our study, lactoferrin triggered submerged biofilm formation by *B. cereus* strains B4158 and ATCC 10987, even though growth was not restored. The underlying mechanism remains to be elucidated.

This study shows that ferric citrate and FeCl_3_ could be used by all *B. cereus* strains and were preferred iron sources. Hemoglobin, hemin, transferrin, ferritin and lactoferrin could also act as iron sources but their use appeared to be highly strain-dependent. The ability of *B. cereus* strains to grow on complex iron sources correlated largely with the genome content, but could not always be linked to specific iron transporter genes present. The ability to use complex iron sources seems to be dictated by the combined presence or absence of more than one functional iron transporting system, rather than one single system. Furthermore, biofilm formation was found to be affected by the type of iron source used, including stimulation of biofilms at liquid-air interphase (FeCl_3_ and Fe citrate) and formation of submerged type biofilms (hemin and lactoferrin). Notably, generation of submerged biofilms was in some cases linked to lack of growth stimulation by the complex iron source tested. To conclude, our results show strain variability in the genome repertoire of iron-transporting systems and differences in efficacy to use complex iron sources for growth and biofilm formation. These features may affect *B. cereus* survival and persistence in specific niches including food processing environments and the human host.

## Materials and methods

### Strains and culturing conditions

Twenty *Bacillus cereus* food isolates from the NIZO culture collection were used in this study (Hayrapetyan et al., 2015a) along with two reference strains *B. cereus* ATCC 10987 and ATCC 14579. To obtain overnight cultures, a loop full with stock cultures stored at −80°C was inoculated into 10 ml LB broth (Miller, MERCK), supplemented with 100 μM 2,2-Bipyridine (Bip) (MERCK) to induce iron starvation, and incubated for 18 h at 30°C with shaking at 200 rpm.

The twenty *B. cereus* food isolates were sequenced by next-generation whole genome sequencing. For eight strains (B4077, B4078, B4080, B4086, B4087, B4147, B4153, B4158), total DNA isolation and sequencing details are described elsewhere (Krawczyk et al., 2015), for the remaining 12 isolates (B4081, B4082, B4083, B4084, B4085, B4088, B4116, B4117 [recently re-classified by NCBI as *Bacillus mycoides* based on ANI typing (Federhen et al., 2016)], B4118, B4120, B4155, B4079) draft genomes were obtained and deposited as described in Hayrapetyan et al. (2016).

### Searching for iron-transporting systems in *B. cereus* genomes

Orthologous groups (OGs; i.e., gene families) were determined using OrthoMCL (Enright et al., 2002). This program uses all-against-all protein BLAST where it groups proteins with more homology within the species than homology with proteins outside the species. In this way orthologs (genes in different species that evolved from a common ancestral gene by speciation) are separated from paralogs (genes related by duplication within a genome). In addition to the 20 newly sequenced genomes of food isolates (Krawczyk et al., 2015; Hayrapetyan et al., 2016), the circular genomes of the two reference strains *B. cereus* ATCC 14579 and ATCC 10987 obtained from the NCBI database, were included. Contigs of the 20 newly sequenced genomes were scaffolded into their presumed correct order using the circular reference genomes as templates.

A database (in MS Excel) was built encompassing information about the location and length of orthologous proteins. Multiple sequence alignment files (MSA) were made (MUSCLE, version 3.8; Edgar, 2004), where the protein sequences within ortholog groups were aligned, to facilitate identification of pseudogenes (encoding incomplete proteins).

A literature search was performed to find known iron-uptake systems for *B. cereus* (Daou et al., 2009; Zawadzka et al., 2009; Hotta et al., 2010). Orthologous groups (OGs) containing the locus tags of these known genes were searched for in the OG table. Furthermore, a key word search was done to find additional iron uptake and storage systems, by searching in the annotation of all genomes for keywords: iron, ferric, ferrous, ferritin.

For relevant identified OGs containing pseudogenes, which are fragments of genes (i.e. truncated, frame-shifted or at the end of contigs), which had been classified by OrthoMCL into separate OGs adjacent on the chromosome, were combined into single OGs representing all the fragments of a single pseudogene.

The RAST automatic annotation of the encoded proteins was manually improved using InterproScan (http://www.ebi.ac.uk/Tools/pfa/iprscan/), NCBI-BLAST (http://blast.ncbi.nlm.nih.gov/http://blast.ncbi.nlm.nih.gov/) and NCBI/Genbank database for the comparison of genes with other species (http://www.ncbi.nlm.nih.gov/).

### Growth and biofilm formation

The growth and biofilm formation on different iron sources was tested in LB (as control), LB supplemented with 600 μM 2,2-Bipyridine (LB+Bip) as iron depleted condition, and in iron-replete conditions using LB+Bip with addition of the following iron sources in final concentrations: FeCl_3_ (250 μM; LB+Bip+FeCl_3_), ferric citrate (250 μM; LB+Bip+Fe citrate), hemoglobin (human, 2.5 μM; LB+Bip+Hb), hemin (4, 8, and 16.5 μM; LB+Bip+Hemin), ferritin (from equine spleen, 0.9 μM; LB+Bip+Ferritin), transferrin (human, partially saturated, 1.5 μM; LB+Bip+Transferrin), and lactoferrin (bovine milk, 0.7 μM; LB+Bip+Lactoferrin). 2,2-Bipyridine, FeCl_3_ and ferric citrate were from MERCK and the remaining iron sources used were obtained from SIGMA. Selected concentrations were adapted from previously reported concentrations used for *B. cereus* (Daou et al., 2009), (Segond et al., 2014), with some optimization for the culturing conditions and strains of this study.

The strains were grown in a 96-well-plate filled with 200 μl LB with or without supplements inoculated with 1% overnight culture. The growth was monitored by measuring the *OD* at 600 nm in SPECTRAmax (model PLUS384) at 30°C, with shaking for 60 s every 5 min. The growth index (GI) for each iron source was calculated as described elsewhere (Daou et al., 2009), by dividing the *OD* at 600 nm reached in LB after 10 h of growth by *OD* reached when grown with the specific iron source.

The biofilms formed in 96-wells-plates inoculated as described above, were measured after 24 h of static incubation at 30°C using the Crystal Violet (CV) assay as described previously (Hayrapetyan et al., 2015a). Washing, staining and de-staining steps were performed using 250 μl of de-mineralized water, 0.1% crystal violet and 70% ethanol, respectively. After de-staining the *OD* was measured at 595 nm. The strain was considered to form a biofilm if in a given condition the *OD* value was higher than 0.1, a threshold value as defined in (Hayrapetyan et al., 2015a).

### Transcriptome analysis to identify iron-responsive genes

For transcriptome analysis RNA was isolated from static liquid cultures of *B. cereus* ATCC 10987 grown in BHI (control), BHI supplemented with 450 μM Bip (BHI+Bip) for iron deplete condition, BHI supplemented with 250 μM FeCl_3_ (BHI+FeCl_3_) and BHI with both Bip and FeCl_3_ (BHI+Bip+FeCl_3_) for iron replete conditions, and the latter to test whether iron supplementation could restore effects evoked by iron starvation induced by Bip. These conditions were based on a previous study in our laboratory showing the role of free iron in biofilm formation (Hayrapetyan et al., 2015a). The samples were taken at exponential growth phase (5 h). RNA was isolated as previously described (Hayrapetyan et al., 2015b). Labeling and hybridization were performed as described elsewhere (Mols et al., 2013). Two independent biological replicates were hybridized on the arrays, each sample was used three times and was labeled with the swapped dyes Cy3 and Cy5.

Custom-made array design for *B. cereus* ATCC 10987 developed by Agilent Technologies (GEO accession number GPL7681; Mols et al., 2010) was used in this study. Microarray scanning and data normalization were performed as previously described (Hayrapetyan et al., 2015b). Genes with more than two fold change in expression and *p* < 0.05 were considered significantly affected. The processed and raw microarray data is deposited in GEO database under accession number GSE74045.

### Statistical analysis

Presented values are averages of at least three independent experiments with standard deviations. The growth was considered recovered if the growth index of the strain on a specific iron source was significantly different from the growth index of the same strain when grown in LB+Bip without iron supplementation. Significance of the growth differences was concluded based on a two-sided student's *t*-test, assuming equal variances and a *P* < 0.01.

## Author contributions

Conceived and designed experiments: TA, MG, and HH. Performed the experiments: HH. Analyzed the data: HH. Performed genomic comparisons: RS. Wrote the paper: HH. All authors read and approved the final manuscript.

### Conflict of interest statement

The authors declare that the research was conducted in the absence of any commercial or financial relationships that could be construed as a potential conflict of interest.
