# Supplementary material for: Comparative Genomics of Iron-Transporting Systems in Bacillus cereus Strains and Impact of Iron Sources on Growth and Biofilm Formation
Source: Front Microbiol. 2016 Jun 8;7:842. doi: 10.3389/fmicb.2016.00842 (PMC4896950; doi:10.3389/fmicb.2016.00842)
Supplement: Supplementary file 1 [file Table1.DOCX]

| Supplementary Table1. Expression of genes encoding iron transporters in *B. cereus* ATCC10987 in iron replete (BHI+Fe and BHI+Bip+Fe) and iron deplete (BHI+Bip) conditions at exponential growth phase (t=5h). Significantly different expression ratios are in bold text and highlighted. Presented values are log_2_ of expression ratios compared to BHI. | | | | | |
| --- | --- | --- | --- | --- | --- |
| **Locus tags in ATCC10987** | **Gene names** | **Gene functions** | **BHI+Bip over BHI** | **BHI+FeCl_3_ over BHI** | **BHI+Bip+Fe Cl_3_ over BHI** |
| BCE2066 | ymfD | hypothetical protein | 1 | 0 | 1.36 |
| BCE2398 | dhbA/entA | 2,3-dihydro-2,3-dihydroxybenzoate dehydrogenase | **8** | 0 | 1.87 |
| BCE2399 | dhbC | isochorismate synthase | **10** | 1 | **3.75** |
| BCE2400 | dhbE | 2,3-dihydroxybenzoate-AMP ligase | **10** | 1 | 2.67 |
| BCE2401 | dhbB | isochorismatase | **10** | 1 | 2.31 |
| BCE2402 | dhbF | non-ribosomal surfactin synthetase SrfAA | **9** | 0 | 2.79 |
| BCE2403 | mbtH | hypothetical protein | **9** | 1 | 2.01 |
| BCE2404 |  | drug resistance transporter, EmrB/QacA family | **9** | 1 | 2.29 |
| BCE2405 | sfp | putative 4'-phosphopantetheinyl transferase | **8** | 1 | 1.45 |
| BCE2406 |  | hypothetical protein | **8** | 1 | 1.72 |
| BCE3767 | yuiI | trilactone hydrolase | **7** | 1 | 1.12 |
| BCE3768 | feuD/yusV | siderophores ABC-transporter, ATP-binding protein FeuC | **6** | 0 | 1.99 |
| BCE3769 | feuC | siderophores ABC-transporter, permease FeuC | **7** | **1** | 2.59 |
| BCE3770 | feuB | siderophores ABC-transporter, permease FeuB | **7** | 0 | 1.34 |
| BCE3771 | feuA | siderophores ABC-transporter, siderophore-binding protein FeuA | **8** | 1 | 1.94 |
| BCE5223 | fhuC | iron-siderophore ABC transporter ATP-binding protein | **4** | 2 | 1.10 |
| BCE5224 | fatC | iron-siderophore ABC transporter permease | **3** | 1 | 0.71 |
| BCE5225 | fatD | iron-siderophore ABC transporter permease | **4** | 1 | 0.98 |
| BCE5226 | fatB | iron-siderophore ABC transporter binding lipoprotein | **4** | 1 | 2.06 |
| BCE1444 | ilsA | iron-regulated Leu-rich surface protein A | **9** | 1 | 1.95 |
| BCE4666 |  | heme-degrading monooxygenase IsdG | **9** | 1 | 2.46 |
| BCE4667 |  | sortase B | **7** | 1 | 1.70 |
| BCE4668 |  | iron compound ABC transporter, ATP-binding protein | **7** | 1 | 2.04 |
| BCE4669 |  | iron compound ABC transporter, permease protein | **6** | 0 | 1.12 |
| BCE4670 |  | iron compound ABC transporter, iron compound-binding protein | **8** | 1 | 1.73 |
| BCE4671 |  | Iron transport-associated protein | **9** | 1 | 2.17 |
| BCE4672 |  | iron transport associated protein | **6** | 1 | 0.15 |
| BCE0683 | fhuD | Iron (III) dicitrate ABC transporter, iron compound-binding protein | **5** | 1 | 1.18 |
| BCE0684 | fecD | Iron (III) dicitrate ABC transporter, permease protein | **3** | 1 | 1.23 |
| BCE0685 | fecC | Iron (III) dicitrate ABC transporter, permease protein | **3** | 1 | 1.28 |
| BCE0686 | fecE | Iron (III) dicitrate ABC transporter, ATP binding protein | **3** | 1 | 1.15 |
| BCE0449 | fhuG | ferrichrome ABC transporter, permease protein | **4** | 1 | 2.48 |
| BCE0450 | fhuB | ferrichrome ABC transporter, permease protein | **4** | 1 | 2.11 |
| BCE0451 | feuA | ferrichrome ABC transporter, ferrichrome-binding lipoprotein | **6** | 1 | 1.83 |
| BCE2283 | yfiY | putative iron compound-binding protein | **6** | 1 | 2.26 |
| BCE3485 | feuA-like | iron compound ABC transporter substrate-bindingprotein FeuA | **6** | 1 | 2.01 |
| BCE3486 | fhuG-like | Ferrichrome transport system permease fhuG | **3** | -1 | 0.86 |
| BCE3487 | fhuB-like | Ferrichrome transport system permease fhuB | **5** | 1 | 2.15 |
| BCE4448 | fepC-lik | iron compound ABC transporter, ATP-bindingprotein | **9** | 1 | 2.16 |
| BCE4449 | fhuG-like | iron compound ABC transporter, permease protein | **7** | 0 | 1.52 |
| BCE4450 | fhuD-like | lipoprotein binding vitamin B12 | **9** | 1 | 2.25 |
| BCE5509 | fepB-like | iron compound ABC transporter, ironcompound-binding protein | **3** | 1 | 1.01 |
| BCE5510 | fepC-like | ferrichrome ABC transporter ATP-binding protein | **3** | 0 | 0.65 |
| BCE5511 | fhuG-like | ferrichrome ABC transporter permease | **4** | 1 | 2.86 |
| BCE5512 | fhuB-like | ferrichrome ABC transporter permease | **4** | 1 | 1.45 |
| BCE0782 | feoB-C | ferrous iron transport protein FeoB, C-terminal domain | 2 | 0 | -0.24 |
| BCE0782 | feoB-N | ferrous iron transport protein FeoB, N-terminal region | 2 | 0 | -0.24 |
| BCE0783 | feoA | ferrous iron transport protein FeoA | 2 | 1 | 0.46 |
| BCE1436 |  | putative iron compound ABC transporter, ironcompound-binding protein | 1 | 0 | 0.68 |
| BCE1437 |  | ABC transporter ATP-binding protein | 2 | -1 | 0.14 |
| BCE1438 |  | iron compound ABC transporter permease | 1 | 0 | 1.58 |
| BCE4965 | feoB | ferrous iron transport protein B | **3** | 2 | 2.11 |
| BCE4966 | feoA | ferrous iron transport protein A | **3** | 2 | 1.99 |
| BCE5191 |  | ferritin-like diiron-binding protein, Dps family | -1 | 1 | 0.02 |
| BCE5196 |  | ferritin-like diiron-binding protein, Dps family | 2 | 1 | 0.10 |
| BCE1087 |  | ferritin-like diiron-binding protein, Dps family | 0 | 0 | 0.06 |
| BCE2092 |  | ferritin-like diiron-binding protein, Dps family | -1 | 0 | -0.51 |
| BCE3134 |  | ferritin-like diiron-binding protein, Dps family | 2 | 1 | 2.26 |
| BCE4160 | fur | iron transport and metabolism transcriptionalregulator (fur) | -1 | 1 | 0.10 |
